# Characteristics of Unplanned Hospital Admissions Among Patients With Cancer Treated at a Comprehensive Cancer Center in Jordan

**DOI:** 10.7759/cureus.77012

**Published:** 2025-01-06

**Authors:** Nour Mustafa, Dima Attili, Farah Alsoud, Nour Alsheikh, Nour Faqeer

**Affiliations:** 1 Pharmacy, King Hussein Cancer Center, Amman, JOR; 2 Pharmacy, University of Jordan, Amman, JOR

**Keywords:** cancer, cancer treatment, hospitalization, infections, patient admission

## Abstract

Purpose: This study describes the characteristics of unplanned hospital admissions of patients with cancer.

Methods: This prospective study was conducted at King Hussein Cancer Center in Amman, Jordan, from March 2023 to August 2023. The study included adult cancer patients who had unplanned hospital admissions, defined as any admission through the emergency department. The follow-up period concluded upon patient discharge, transfer to palliative service, or death. Patients’ medical charts were reviewed to collect baseline characteristics and data related to the admission, including the cause of admission, length of stay (LOS), and hospitalization outcomes.

Results: During the study period, a total of 2435 admissions were included. The mean age of the admitted patients was 56.9 years ± 14.7 SD, with 49.8% (n = 1212) being male. The most common cancer types were gastrointestinal, hematological, and breast cancers, accounting for 20.9% (n = 508), 20.5% (n = 499), and 20.0% (n = 488) of the admissions, respectively. Among the included admissions, 84.4% were directed to the Medical Oncology service. The most common causes of admission were infection (31.5%), followed by complications from cancer treatment (10.4%). Among the included patients, 87% (n = 2118) were discharged home after a median LOS of five days (range 1-66), 3.3% (n = 81) were transferred to palliative care, and 9.7% (n = 235) died. Infection was associated with the longest LOS compared to other causes of admission.

Conclusion: Among the unplanned hospital admissions of cancer patients, infections and complications from cancer treatment were the most common causes. Further studies are warranted to assess the preventability of these admissions.

## Introduction

Cancer poses a significant global health challenge and is the most common cause of mortality in the United States [1]. In Jordan, cancer incidence is rising rapidly and has become a major cause of morbidity and mortality [2]. This trend reflects increasing public health challenges observed across the Middle East region, including Jordan, where lifestyle changes, such as increased smoking rates among the population and limited access to screening and preventive measures, have contributed to the growing incidence of cancer.

Over the past decade, advancements in early detection, surgical procedures, and targeted therapies have improved treatment responses and overall survival rates [1]. Nevertheless, the medical needs of cancer patients remain complex, requiring a comprehensive and personalized approach to care. This care extends beyond diagnosis and treatment to include a thorough assessment of symptom management, psychological well-being, and supportive care. Collaborative efforts among healthcare providers are pivotal in ensuring that medical needs are not only met but also continually refined to enhance the quality of life for those affected by cancer. Cancer patients are at high risk for complications due to the complexity of their disease, challenges associated with chemotherapy and/or targeted therapies, and complications arising from underlying comorbidities. This complexity increases the likelihood of hospital admissions and places a substantial burden on the healthcare system [3].

Unplanned hospital admissions for cancer patients can arise from various factors. Oncologic emergencies, such as hypercalcemia and thrombosis, constitute a significant contributor to these admissions [4]. Complications from cancer treatment, such as gastrointestinal side effects of chemotherapy and neutropenic fever, are also common causes of unplanned hospital stays [5]. Infection is another frequent cause of admission, with origins ranging from infected malignant wounds to catheter-related infections, making cancer patients particularly vulnerable. A foundational understanding of the nature of these admissions is warranted to determine the preventability of unnecessary hospitalizations, aiming to conserve valuable resources and redirect them toward advancements in care.

Several studies have evaluated hospital admissions of patients with cancer [3, 6-11]. However, most of these studies have been retrospective in nature and focused on specific tumor types or causes of admission, limiting the understanding of the broader admission landscape [6, 12-18]. For example, a study conducted by Chan et al. exclusively explored admissions related to drug-related problems, highlighting a narrow focus on a specific aspect of cancer care [19]. Furthermore, there is limited evidence evaluating hospital admissions in our region. In light of these gaps, we conducted a prospective study to assess the characteristics and predictors of unplanned hospital admissions at a comprehensive cancer center in Jordan.

This article was previously presented as a meeting abstract at the 2024 KHCC Research Conference on October 3, 2024, and as a poster at the ACCP Virtual Poster Symposium on May 23, 2024.

## Materials and methods

This prospective observational study was conducted at King Hussein Cancer Center (KHCC). KHCC is a 350-bed, internationally accredited cancer center located in Amman, Jordan. It provides comprehensive care to adult and pediatric patients from Jordan and the Middle East with all types of cancer. This study was performed in line with the principles of the Declaration of Helsinki. Ethics approval was granted by the institutional review board on March 8, 2023, under approval number 22 KHCC 176. A waiver of consent was requested due to the minimal risk posed by the collected data to the patients, and the waiver was deemed not to adversely affect the rights and welfare of the subjects. Furthermore, the research could not have been conducted without the waiver.

Data were collected over a six-month period from March 2023 to the end of August 2023. The study included cancer patients aged 18 years and older who were admitted to the hospital through the emergency department with an unplanned admission diagnosis. The follow-up period concluded upon the patient's discharge, transfer to palliative service, or death. Patients admitted for planned surgery, elective chemotherapy administration, or elective procedures were excluded. Additionally, adult cancer patients admitted to the hospice and palliative care service, those aged 18 years and older under the pediatric service, and patients readmitted within one month of their initial admission were excluded.

Patients were identified based on the inclusion and exclusion criteria by daily reviewing the hospital admission list. The Computerized Patient Record System (CPRS), the electronic documentation system at KHCC, was used to gather data on patients' baseline characteristics and admission information. This study was conducted prospectively, with patients followed daily from the time of admission until their discharge, death, or transfer to palliative and hospice care. Data were collected during their hospital stay. The patients’ baseline characteristics upon admission included age, gender, type of malignancy, metastatic status of the malignancy, whether patients were undergoing active antineoplastic treatment, and the number of chronic medications taken (i.e., medications taken for any chronic illness other than cancer). Additionally, data related to the admission, including reason for admission, discharge date, length of hospital stay (LOS), and hospitalization outcomes, which included all-cause mortality, transfer to palliative and hospice care, and discharge home, were also collected.

The causes of admissions were classified according to the admission diagnosis included in the admission note by the treating physicians in the CPRS. These included infections, cancer treatment complications (vomiting, neutropenic fever, mucositis, medication toxicity), neurological causes (seizures, decreased level of consciousness, brain metastasis, stroke), electrolyte disturbances (hypercalcemia, hypokalemia, hypomagnesemia, hyponatremia), renal failure, pain crisis, hematological causes (non-gastrointestinal bleeding, anemia, and thrombocytopenia), gastrointestinal causes (nausea, vomiting, acute pancreatitis, diarrhea, gastrointestinal obstruction, gastrointestinal bleeding), cardiac causes (heart failure, atrial fibrillation, myocardial infarctions), and respiratory causes (asthma, pleural effusions, and chronic obstructive pulmonary disease exacerbation). We utilized classifications similar to those used in a study by Zhuang et al. [6].

Sample size estimation

The sample size for the study was determined based on previous similar studies, which reported sample sizes ranging between 400 and 2000 admissions. To ensure sufficient power and precision in our statistical analyses, we decided to include at least 2000 admissions in the study.

Statistical analysis

Frequencies and percentages were used to characterize categorical variables, while measures of central tendency (mean and median) and measures of dispersion (standard deviation and range) were used to describe continuous variables. The relationship between categorical variables was assessed using the chi-square test or Fisher’s exact test as an alternative. The Mann-Whitney U-test (non-parametric) was used to examine associations between two groups of patients, and the Kruskal-Wallis H-test (non-parametric) was used to examine associations among three or more groups. These tests were selected due to the non-normal distribution of the data. Multivariate analysis was performed for the significant factors using logistic regression analysis for binary outcomes and multinomial logistic regression for outcomes with more than two categories. A P-value of ≤0.05 was considered statistically significant. All analyses were performed using SAS version 9.4 (SAS Institute Inc., Cary, North Carolina).

## Results

During the study period, 2435 admissions were included. The mean age of the patients was 56.9 years (±14.7), with 49.8% (n = 1212) being male. The most common malignancies were gastrointestinal, hematological, and breast cancers. Patients’ baseline characteristics are summarized in Table 1. Among the included admissions, 768 (31.5%) were related to infections, followed by complications from cancer treatment, which accounted for 254 (10.4%) admissions. Table 2 demonstrates the distribution of admission diagnoses.

**Table 1:** Baseline characteristics of 2435 unplanned admissions to the hospital between March 10, 2023, and the end of August 2023

Variable	Frequency (%)
Gender	
Male	1212 (49.8%)
Female	1223 (50.2%)
Malignancy type	
Gastrointestinal	508 (20.9%)
Hematological	499 (20.5%)
Breast	488 (20.0%)
Lung	273 (11.2%)
Genitourinary	199 (8.2%)
Gynecological	153 (6.3%)
Head and neck	125 (5.1%)
Brain tumors	101 (4.1%)
Sarcoma	68 (2.8%)
Melanoma	14 (0.6%)
Unknown	7 (0.3%)
Metastatic (Stage IV)	
Yes	1819 (74.7%)
No	616 (25.3%)
On active treatment	
Yes	1248 (51.3%)
No	1187 (48.7%)
Treatment types (N=1248)	
Antineoplastic	1088 (87.2%)
Radiotherapy	62 (5.0%)
Concurrent chemoradiotherapy	98 (7.9%)
Chronic medications	
None	701 (28.8%)
1-2	668 (27.4%)
3-5	665 (27.3%)
More than 5	401 (16.5%)
Comorbidities	
One	604 (24.8%)
More than 1	1170 (48.0%)
None	661 (27.1%)
Admitting service	
Medical oncology	2065 (84.8%)
Critical care	172 (7.0%)
Surgical	126 (5.2%)
Bone marrow transplantation	72 (2.9%)

**Table 2:** Causes of 2435 unplanned hospital admissions between March 10, 2023, and the end of August 2023

Admission Diagnosis	Frequency (%)
Infections	768 (31.5 %)
Cancer treatment complications	254 (10.4 %)
Neurological causes	244 (10.0 %)
Electrolytes disturbances	199 (8.1 %)
Renal failure	166 (6.8 %)
Pain crisis	152 (6.2 %)
Hematological causes	150 (6.2 %)
Gastrointestinal causes	218 (9.0 %)
Cardiovascular causes	118 (4.8 %)
Respiratory causes	132(5.4 %)
Others	34 (1.4 %)

Among the included patients, 2118 (87.0%) were discharged home, 81 (3.3%) were transferred to palliative care, and 235 (9.7%) died. One patient was not discharged during the study period. The median LOS for patients who were discharged home was five days (range: 1-66). Patients admitted with infections had a longer median LOS of six days (range: 1-66) compared to five days (range: 1-54) for those admitted with other causes. The median LOS for patients who died was 11 days (range: 1-151), and the median time to transfer to the palliative and hospice care service was eight days (range: 2-74).

In multivariate analysis, patients receiving active antineoplastic treatment had a lower likelihood of being admitted for infection (odds ratio (OR) 0.79; 95% confidence interval (CI) 0.66-0.94, P = 0.007). Hematological malignancies were significantly associated with infection-related admissions (OR 1.77; 95% CI 1.44-2.17, P = 0.0001). The univariate analysis is presented in Table 3. Admissions due to complications from cancer treatment were also significantly associated with hematological malignancies and patients receiving active antineoplastic treatment. In contrast, male gender, metastatic disease, and increasing age were inversely associated with admissions due to complications from antineoplastic treatment. The univariate analysis and the multivariate logistic regression analysis of variables associated with admissions due to cancer treatment complications are presented in Tables 4, 5.

**Table 3:** Univariate analysis for admission reason (infections versus others) (N = 2435) Statistical test used: the Mann-Whitney U-test (non-parametric) was used to examine the association between two groups of patients, and the Kruskal-Wallis H-test (non-parametric) was used to examine the association between three or more groups.

Variable	Level	Total	Admission Reason		P-value
			Infection	Others	
Hospital outcome (1 patient was not discharged throughout the study period)	Transfer palliative	81 (3.3%)	18 (22.2%)	63 (77.8%)	0.002
	Death	235 (9.7%)	95 (40.4%)	140 (59.6%)	
	Discharge home	2118 (87.0%)	655 (30.9%)	1463 (69.1%)	
Status	Alive	2200 (90.3%)	674 (30.6%)	1526 (69.4%)	0.002
	Dead	235 (9.7%)	95 (40.4%)	140 (59.6%)	
Gender	Male	1212 (49.8%)	395 (32.6%)	817 (67.4%)	0.267
	Female	1223 (50.2%)	373 (30.5%)	850 (69.5%)	
Malignancy type	Hematological	499 (20.5%)	207 (41.5%)	292 (58.5%)	0.000
	Solid tumor	1936 (79.5%)	561 (29.0%)	1375 (71.0%)	
Metastatic	Metastatic	1819 (74.7%)	568 (31.2%)	1251 (68.8%)	0.567
	Not metastatic	616 (25.3%)	200 (32.5%)	416 (67.5%)	
On active treatment	On active	1248 (51.3%)	366 (29.3%)	882 (70.7%)	0.016
	Not on active	1187 (48.7%)	402 (33.9%)	785 (66.1%)	
Comorbidities	None	661 (27.1%)	210 (31.8%)	451 (68.2%)	0.881
	One or more comorbidity	1774 (72.9%)	558 (31.5%)	1216 (68.5%)	
Chronic medications	1-2	668 (27.4%)	218 (32.6%)	450 (67.4%)	0.663
	3-5	665 (27.3%)	198 (29.8%)	467 (70.2%)	
	More than 5	401 (16.5%)	125 (31.2%)	276 (68.8%)	
	Zero	701 (28.8%)	227 (32.4%)	474 (67.6%)	
Length of stay (median (range)), days			6 (1-66)	5 (1-54)	0.000
Age (median (range))			58 (19-91)	58 (18-93)	0.503

**Table 4:** Univariate analysis for admission reason (cancer treatment complications versus others causes of admissions) (N = 2435) Statistical test used: the Mann-Whitney U-test (non-parametric) was used to examine the association between two groups of patients, and the Kruskal-Wallis H-test (non-parametric) was used to examine the association between three or more groups.

Variable		Total	Admission Reason		P-value
			Admissions Due to Cancer Treatment Complications	Other Causes	
Hospital outcome (one patient was not discharged throughout the study period)	Transfer palliative care	81 (3.3%)	3 (3.7%)	78 (96.3%)	0.000
	Death	235 (9.7%)	6 (2.6%)	229 (97.4%)	
	Discharge home	2118 (87.0%)	238 (11.2%)	1880 (88.8%)	
Status	Alive	2200 (90.3%)	241 (11.0%)	1959 (89.0%)	0.000
	Dead	235 (9.7%)	6 (2.6%)	229 (97.4%)	
Gender	Male	1212 (49.8%)	100 (8.3%)	1112 (91.7%)	0.002
	Female	1223 (50.2%)	147 (12.0%)	1076 (88.0%)	
Malignancy type	Hematological	499 (20.5%)	102 (20.4%)	397 (79.6%)	0.000
	Solid tumor	1936 (79.5%)	145 (7.5%)	1791 (92.5%)	
Metastatic	Metastatic	1819 (74.7%)	156 (8.6%)	1663 (91.4%)	0.000
	Not metastatic	616 (25.3%)	91 (14.8%)	525 (85.2%)	
On active treatment	On active	1248 (51.3%)	221 (17.7%)	1027 (82.3%)	0.000
	Not on active	1187 (48.7%)	26 (2.2%)	1161 (97.8%)	
Comorbidities	More than 1	1170 (48.0%)	76 (6.5%)	1094 (93.5%)	0.000
	None	661 (27.1%)	93 (14.1%)	568 (85.9%)	
	1 comorbidity	604 (24.8%)	78 (12.9%)	526 (87.1%)	
Chronic medications	1-2	668 (27.4%)	84 (12.6%)	584 (87.4%)	0.000
	3-5	665 (27.3%)	47 (7.1%)	618 (92.9%)	
	More than 5	401 (16.5%)	22 (5.5%)	379 (94.5%)	
	Zero	701 (28.8%)	94 (13.4%)	607 (86.6%)	
Length of stay (median(range), days			5 (1-39)	5 (1-66)	0.861
Age (median (range))			51 (18-80)	59 (18-93)	0.000

**Table 5:** Logistic regression analysis for admission reason (cancer treatment complications versus others)

Effect	Odds Ratio	95% Confidence Limits		P-value
Malignancy type (hematological versus solid tumor)	2.830	2.072	3.866	<0.0001
Gender (male versus female)	0.612	0.453	0.825	0.0013
Metastatic (metastatic vs non-metastatic)	0.468	0.344	0.639	<0.0001
On active treatment (receiving versus not receiving)	10.118	6.607	15.494	<0.0001
Comorbidities (none versus yes)	1.076	0.772	1.499	0.6658
Age	0.972	0.962	0.983	<0.0001

Multivariate analysis also revealed that being alive at the end of hospitalization was inversely associated with metastatic disease, increasing age, and increasing LOS. Conversely, receiving active antineoplastic treatment was significantly associated with survival at the end of hospitalization. The univariate analysis and the multivariate logistic regression analysis of factors associated with survival at the end of hospitalization are presented in Tables 6, 7.

**Table 6:** Univariate analysis for survival at the end of hospitalization (N = 2435) Statistical test used: the Mann-Whitney U-test (non-parametric) was used to examine the association between two groups of patients, and the Kruskal-Wallis H-test (non-parametric) was used to examine the association between three or more groups.

Variables	Level	Total	Status		P-value
			Alive	Dead	
Gender	Male	1212 (49.8%)	1090 (90.0%)	121 (10.0%)	0.576
	Female	1223 (50.2%)	1109 (90.7%)	114 (9.3%)	
Malignancy type	Hematological	499 (20.5%)	454 (91.2%)	44 (8.8%)	0.487
	Solid	1936 (79.5%)	1745 (90.1%)	191 (9.9%)	
Metastatic	Metastatic	1819 (74.7%)	1614 (88.8%)	204 (11.2%)	0.000
	Not metastatic	616 (25.3%)	585 (95.0%)	31 (5.0%)	
On active treatment	On active	1248 (51.3%)	1158 (92.8%)	90 (7.2%)	0.000
	Not on active	1187 (48.7%)	1041 (87.8%)	145 (12.2%)	
Comorbidities	None	661 (27.1%)	608 (92.1%)	52 (7.9%)	0.070
	Yes	1774 (72.9%)	1591 (89.7%)	183 (10.3%)	
Chronic medications	1-2	668 (27.4%)	593 (88.8%)	75 (11.2%)	0.200
	3-5	665 (27.3%)	598 (89.9%)	67 (10.1%)	
	More than 5	401 (16.5%)	363 (90.5%)	38 (9.5%)	
	Zero	701 (28.8%)	645 (92.1%)	55 (7.9%)	
Length of stay (median (range)), days			5 (1-74)	11 (1-151)	0.000
Age (median (range))			58 (18-93)	60 (22-89)	0.028

**Table 7:** Logistic regression analysis for survival at the end of hospitalization

Effect	Odds Ratio	95% Confidence Limits		P-value
Metastatic (metastatic versus non-metastatic)	0.349	0.228	0.532	<0.0001
On active treatment (on active treatment versus not on active treatment)	1.786	1.332	2.396	0.0001
Length of stay	0.917	0.903	0.931	< .0001
Age	0.987	0.977	0.997	0.0118

## Discussion

In this study, we investigated the characteristics of unplanned hospital admissions of cancer patients, including both demographic and clinical characteristics, as well as hospitalization outcomes. Our findings provide valuable insights into the predictors of these admissions, shedding light on potential areas for intervention and improvement in clinical management. The mean age of our patient cohort was notably different from that reported in previous studies. In our study, the mean age of admitted patients was 56.9 years, with nearly equal distribution between genders. This contrasts with findings from other studies, which indicated higher admissions among male patients and a median age of 69.2 years, particularly in the context of avoidable admissions [20].

In the present study, gastrointestinal, hematological, and breast malignancies were the predominant cancer types among admitted patients, likely influenced by the complexities of their treatment regimens and the incidence rates within Jordan. Notably, breast cancer is the most prevalent malignancy in Jordan and ranks as the third leading cause of mortality after lung and colorectal malignancies [21]. Similar findings have been reported in other studies. For instance, a study conducted in Italy by Numico et al. examined 454 unplanned admissions over 16 months, revealing gastrointestinal cancer as the most frequent, followed by lung and hematological malignancies [3]. Likewise, a retrospective study conducted in Singapore involving 1624 patients over six months highlighted gastrointestinal tumors as leading to the highest number of hospital admissions [6]. Whitney et al. retrospectively found that gastrointestinal tumors were associated with the highest number of admissions over a three-year period post-diagnosis [7]. Rocque et al.'s retrospective analysis similarly identified gastrointestinal, lung, and breast cancers as frequent causes of unplanned admissions [10]. In a comparative analysis by Numico et al., lung, breast, and gastrointestinal cancers were the most common malignancies in hospital admissions compared to non-cancer patients [9]. Notably, while several retrospective studies have focused on specific cancer types such as lung, head and neck, and colon cancers, our study included a broader spectrum of cancer types [12-15, 17].

In our study, almost two-thirds of admitted patients had metastatic disease, and approximately half had multiple comorbidities. This is expected, given that patients with advanced disease and metastasis are more prone to developing complications related to tumor growth in the metastatic site. Moreover, the presence of medical comorbidities increases the risk of cancer-related complications, with greater severity upon their occurrence. Our univariate analysis further demonstrated that the presence of metastasis was associated with death at the end of hospitalization. This finding was supported by the multivariate analysis, which revealed that having metastatic disease was linked to a 65% lower likelihood of survival by the end of hospitalization. In our analysis of all-cause mortality, this correlation is likely because patients eligible for active antineoplastic treatment tend to be in better overall health.

Notably, approximately one-third of admissions were attributed to infections, highlighting the significant burden of infectious complications in cancer patients. This finding underscores the importance of infection prevention and control within oncology care settings. Furthermore, the longer LOS observed in patients admitted with infections highlights the clinical impact of these admissions on healthcare resource utilization and patient outcomes. Infections were also identified as the most common reason for admission in previous studies [6-7, 9]. In our study, distinguishing infections associated with chemotherapy from those unrelated to chemotherapy was challenging, as the classification of admission diagnoses was solely based on the diagnoses documented in physicians' notes. Additionally, some patients may have developed infections while not actively receiving chemotherapy. Therefore, all infections were classified under the infectious category for the reason for admission. However, neutropenic fever was categorized as a complication of chemotherapy, rather than an infection, given its clear and direct association with chemotherapy.

Cancer symptoms were also the predominant cause of admission in an Italian study and a retrospective study of six months’ hospital admissions [3, 18]. Nausea, vomiting, pain, and fever were among the most common presentations in a retrospective analysis [8] examining the effect of outpatient chemotherapy on unplanned hospital admissions among patients, with a reported median LOS of five days, similar to our study. In our study, complications from cancer treatment also constituted a notable proportion of admissions, reflecting the toxicity and side effects associated with cancer therapies. Further research is warranted to study the underlying factors driving the preventability of such admissions in high-risk patient subgroups.

The median LOS was five days, with a longer LOS for patients who died, which was almost doubled. This could be attributed to the fact that these patients were sicker and required longer hospitalization time. Studies conducted in Brazil demonstrated longer hospitalization for patients with metastatic disease and comorbidities [9, 22]. Cancer patients often require supportive measures upon discharge, such as oxygen devices, antibiotics, and multiple appointments, all of which could delay discharge and thereby increase LOS. Our study showed that patients admitted with infections had a longer LOS compared to other causes of admission. This may be related to the need for extended courses of antibiotics.

Our analysis revealed that patients not receiving active cancer treatment and patients with hematological malignancies were independently associated with admissions due to infections. This underscores the complexity of hematological malignancies and their susceptibility to opportunistic infections compared to solid tumors and the vulnerability of those patients to infectious complications due to immunocompromised status, highlighting the need for comprehensive monitoring and prophylactic measures for this cohort to prevent such admissions. Patients undergoing active antineoplastic treatment faced a nearly tenfold increased risk of admission due to complications related to cancer treatment. Specifically, those with hematological malignancies had a 2.8 times higher likelihood of such admissions, attributed to the more intensive chemotherapy regimens they receive, which contribute to treatment complications. Conversely, patients with metastatic disease had a 50% lower incidence of admission due to complications from cancer treatment, likely because they typically undergo treatment with palliative intent to minimize toxicity and complications. Additionally, male gender was associated with a 40% reduction in the odds of admission for cancer treatment complications. Furthermore, there was an inverse relationship between patient age and the likelihood of admission for this reason. This suggests that clinical presentations should be considered to inform management decisions, prompting further investigation into how these variables impact treatment plans and outcomes. Furthermore, multivariate analysis of survival at the end of hospitalization indicated that increasing age, metastatic disease, and longer LOS were associated with reduced survival, while receiving active antineoplastic treatment was positively correlated with survival. This finding underscores the importance of active treatment in improving patient outcomes and highlights the need for intensive supportive therapy for this patient population to maximize treatment outcomes and reduce complications.

The findings of this study provide valuable insights for improving clinical practice and optimizing resource use in the inpatient setting. This study supports the development of proactive preventive measures and quality improvement projects related to infectious disease services and enhances the hospital's antimicrobial stewardship program. Furthermore, it identifies populations more likely to be admitted with cancer treatment complications, highlighting opportunities for improvement in preventive measures, such as pre- and post-chemotherapy medications, to reduce the risk of hospital admissions.

The strengths of our study include its prospective design, which minimized the risk of missing data. Furthermore, this study included a relatively large cohort of patients with a variety of tumors compared to previous studies. However, we acknowledge several limitations. First, it was conducted at a single cancer center, which might limit the generalizability of our findings. Second, we did not investigate some important risk factors that might affect admissions, such as time from diagnosis to admission and stage at diagnosis. Additionally, we lacked a method to objectively assess admission diagnoses, relying solely on the diagnoses recorded in patients’ computerized records. Lastly, we did not study the preventability of unplanned admissions. Future studies incorporating the observed characteristics and clinical data of admissions, along with longitudinal follow-up, are warranted to validate our findings and explain the causal relationships underlying such admissions.

## Conclusions

In this study, unplanned admissions were more prevalent among patients with metastatic disease and those with hematological, breast, or gastrointestinal tumors. Among the causes of unplanned admissions, infections were the most frequent, often resulting in longer hospital stays compared to other admission reasons. These findings underscore the importance of enhancing infection management practices. Further investigation is warranted to assess the preventability of these admissions, explore readmissions, and evaluate their cost implications on the healthcare system, aiming to optimize resource allocation and cost-saving while enhancing clinical practice. Our study provides valuable insights into predicting admissions related to infections and cancer treatment complications in a relatively large cohort of cancer patients, highlighting opportunities for interventions to improve patient outcomes.
